# Identifying Community-Based Needs of Older Family Caregivers: A Systematic Review & Meta-Synthesis

**DOI:** 10.1093/geroni/igaf122.2977

**Published:** 2025-12-31

**Authors:** Arielle Galinsky, Clara Sorkin, Elizabeth Marfeo

**Affiliations:** Tufts University Health & Productive Aging Lab, Medford, Massachusetts, United States; Tufts University Health & Productive Aging Lab, Medford, Massachusetts, United States; Tufts University, Medford, Massachusetts, United States

## Abstract

The demand for family caregiving in the United States is significant, with millions providing unpaid care for individuals with dementia, often encountering substantial emotional, financial, and physical challenges. Expanding home and community-based support services is essential to reducing family caregiver burden, improving the quality of life for individuals with dementia, facilitating aging in place, and decreasing dependence on long term institutional care. A systematic review and meta-synthesis of qualitative research was conducted to examine unmet needs among older adult family caregivers of individuals with dementia. A comprehensive search strategy was implemented across multiple electronic databases spanning medical, psychological, social, and nursing sciences from 2014 to 2025. A total of 33 studies met inclusion criteria, representing perspectives from 879 family caregivers aggregated across all studies included. Key themes identified include: (1) the influence of cultural and social factors on coping strategies and access to support; (2) the evolving dyadic nature of caregiving as dementia progresses; and (3) the prevalence of unmet emotional and mental health needs. These findings provide a robust evidence base for informing the development of targeted home and community-based support programs, as well as future research initiatives aimed at addressing the diverse needs of older adult family caregivers of individuals with dementia.

